# Ossifying fibroma of the nasal cavity: Case report and literature review

**DOI:** 10.1016/j.ijscr.2025.111030

**Published:** 2025-02-11

**Authors:** Mohammed Almusallam, Mohammed Asiri, Fahad Alwadi, Bader Alsaab, Mohammad Almahdi

**Affiliations:** a Division of Otolaryngology – Head and Neck Surgery, Department of Surgery, King Abdulaziz Medical City, Ministry of the National Guard - Health Affairs, Riyadh, Saudi Arabia; b King Abdullah International Medical Research Center, Riyadh, Saudi Arabia; c College of Medicine, King Saud bin Abdulaziz University for Health Sciences, Riyadh, Saudi Arabia; d Department of Anatomic Pathology, King Abdulaziz Medical City, Riyadh, Saudi Arabia

**Keywords:** Ossifying fibroma, Nasal cavity, Neoplasm, Paranasal sinuses, Stroma

## Abstract

**Introduction and importance:**

Ossifying fibroma is a benign, encapsulated, or demarcated neoplasm consisting of varying amounts of cementum-like tissue or bone within a fibrous tissue stroma. It commonly affects patients aged 20 to 40 years, with a predominance in females; however, it can also affect children and adolescents. In this case report, we will present a rare instance of ossifying fibroma in the nasal cavity.

**Case presentation:**

A 25-year-old female, a known case of B-cell lymphoma diagnosed in 2022 and treated with chemotherapy, was referred to ENT for an abnormal CT finding. The CT showed a complex lesion in the left nasal cavity with peripheral calcification and extension to the left maxillary sinus, left frontal sinus, and ethmoidal air cells. The remaining paranasal sinuses were well-aerated, and no associated bony destruction was found.

**Clinical discussion:**

The appearance of ossifying fibroma lesions in the nasal cavity is uncommon. In the literature, most cases of ossifying fibroma are typically found in sites such as the mandible and maxilla. It usually present as a painless bony mass that can give rise to symptoms depending on the site affected. The mainstay management is primarily surgical. It is suggested that without complete removal of the lesion, recurrence can occur between 6 months to 7 years after resection.

**Conclusion:**

Ossifying fibroma is a benign bony lesion that most commonly affects the mandible and maxilla. It primarily occurs in females during their second to fourth decades of life. Such lesions are usually asymptomatic until they grow large enough to cause symptoms. Management for such tumor is primarily surgical to avoid recurrence.

## Highlights

• Ossifying fibromas usually occur in sites such as the mandible. However, their appearance in the nasal cavity is uncommon.
• Ossifying fibromas are often asymptomatic until they grow large enough to produce noticeable swelling and deformity.
• The management of ossifying fibroma is primarily surgical due to the tendency of these lesions to recur.

## 1 Introduction

The definition of ossifying fibroma is that it is a benign, encapsulated, or demarcated neoplasm consisting of varying amounts of cementum-like tissue or bone within a fibrous tissue stroma [1,2]. This type of benign tumor primarily consists of bone that is enveloped by fibrous connective tissue. It is usually characterized as a slowly growing benign neoplasm that predominantly affects the jaw and mandible [3].

Ossifying fibroma most commonly affects patients aged 20 to 40 years, with a predominance in females; however, it can also affect children and adolescents [1]. While the mandible is the most commonly affected site, this benign neoplasm can also involve other areas, including the maxilla, facial and cranial bones, ethmoid, sphenoid, frontal, and temporal bones [4,5]. Patients with ossifying fibroma may present with an expanding, round or ovoid, painless bone mass that, depending on the affected site, can give rise to different symptoms. As the jaw is the most documented site in the literature, it can cause displacement of the roots of adjacent teeth [6].

In this case report, we will present a rare instance of ossifying fibroma in the nasal cavity. Although some cases of ossifying fibroma in the nasal cavity have been reported [7,8], its incidence compared to sites like the mandible, maxilla, or paranasal sinuses remains low.

## 2 Methods

This work was completed in line with the SCARE criteria [9].

### 2.1 Case presentation

**Fig. 1:**
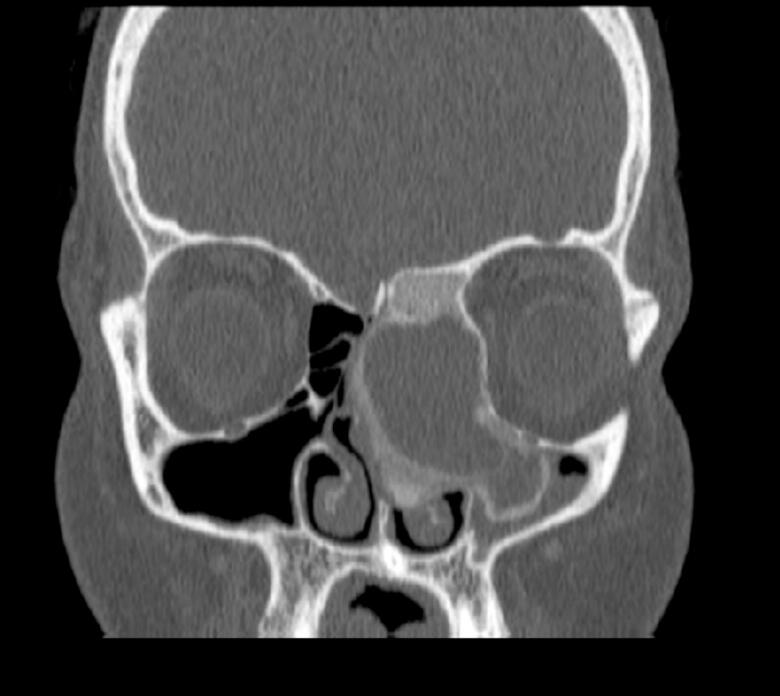
Coronal view Computed Tomography (CT) of paranasal sinuses showing left sided nasal cavity lesion with peripheral calcification extending to left maxillary sinus.

**Fig. 2:**
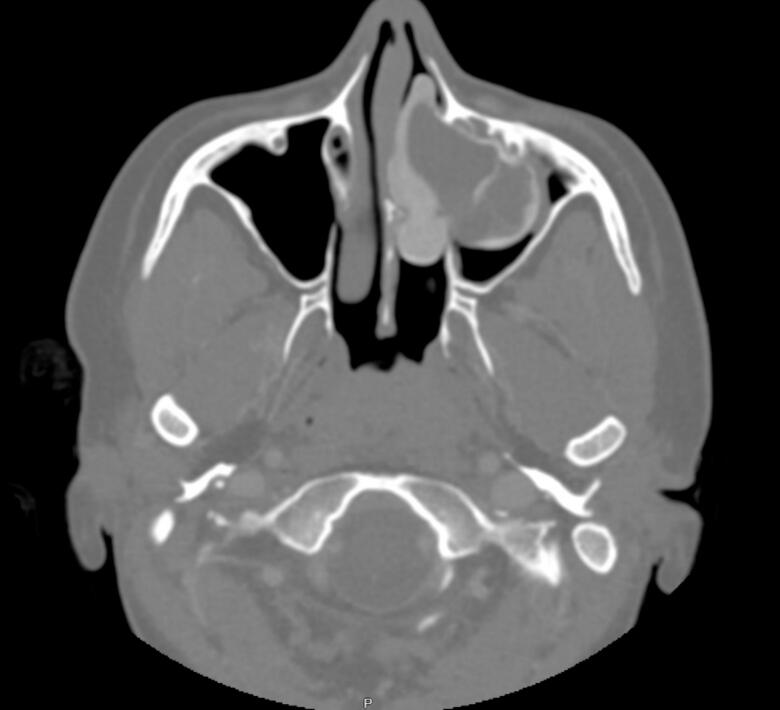
Axial view Computed Tomography (CT) of paranasal sinuses showing left sided nasal cavity lesion with peripheral calcification extending to left maxillary sinus.

**Fig. 3:**
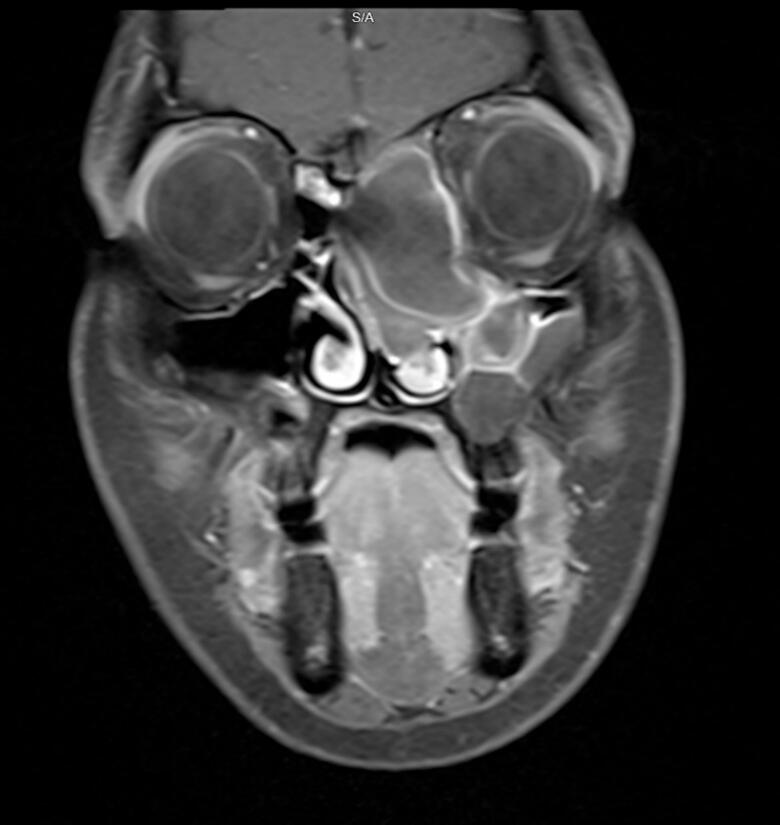
Coronal view T1 with contrast Magnetic Resonance Imaging (MRI) of paranasal sinuses showing expansion of the left nasal cavity and ethmoid the air cells with large cystic and solid component mass.

A 25-year-old female, a known case of B-cell lymphoma diagnosed in 2022 and treated with chemotherapy, was referred to ENT for an abnormal CT finding. The CT showed a complex lesion in the left nasal cavity with peripheral calcification and extension to the left maxillary sinus, left frontal sinus, and ethmoidal air cells (Fig. 1, Fig. 2). The remaining paranasal sinuses were well-aerated, and no associated bony destruction was found. A brain MRI revealed a hypo-intense soft tissue lesion involving the left nasal cavity and ethmoid air cells, demonstrating homogeneous contrast enhancement (Fig. 3).

**Fig. 4:**
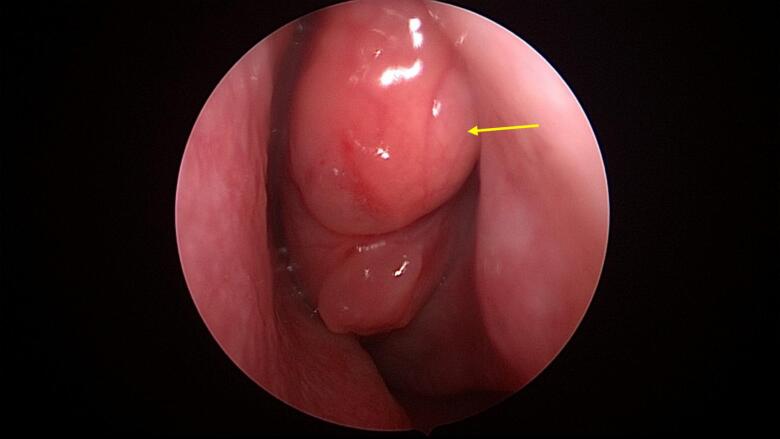
Nasal endoscopy of the left nasal cavity showing a mass occupying the left nasal cavity.

The patient was admitted under ENT to be scheduled for nasal endoscopy and biopsy. During the procedure, examination of the nasal cavity and/or postnasal space was performed, along with an excisional biopsy of the left nasal mass. Using a 0-degree rigid scope of 4 mm, the right side of the nasal cavity was found to be patent and clear. On the left side, a mass occupied the nasal cavity and osteomeatal complex, along with a left septal spur (Fig. 4). Multiple biopsies were taken from the nasal mass, which originated from the middle turbinate, and were placed in formalin for histopathology as well as sent fresh. Following this, the mass was completely excised, leaving only a thin layer of the medial part of the middle turbinate intact.

Next, using a debrider, the entire nasal mass and cavity were debrided, and the maxillary sinus mass was also completely excised. After this, the maxillary sinus appeared clean, and there was already a wide maxillary antrostomy that was further widened. Using suction coagulator 25 spray mode, cauterization of areas of bleeding was done specially the sphenopalatine area.

We then shifted to a 30-degree scope to examine the frontal sinus. Minimal tissue was found, which was also removed and sent for histopathology. The frontal sinus was patent and minimally widened. Irrigation was performed inside the frontal sinus, which was clear. Afterward, surgical snow was placed on the raw areas, and the procedure was concluded.

The pathology report for the biopsies was described as follows:

Part 1 (Left maxillary mass): This section consisted of bony tissue measuring 2.0 × 1.5 × 0.5 cm, with attached friable, tan-brown soft tissue measuring an aggregate of 0.8 × 0.5 × 0.2 cm. Biopsies were taken from both the left middle turbinate mass and the left maxillary mass, with both showing histopathological features of psammomatoid ossifying fibroma.
Part 2 (Left middle turbinate mass): This section consisted of bony tissue measuring 2.0 × 2.0 × 1.0 cm, with attached friable, hemorrhagic soft tissue measuring an aggregate of 1.0 × 0.8 × 0.5 cm.

**Fig. 5:**
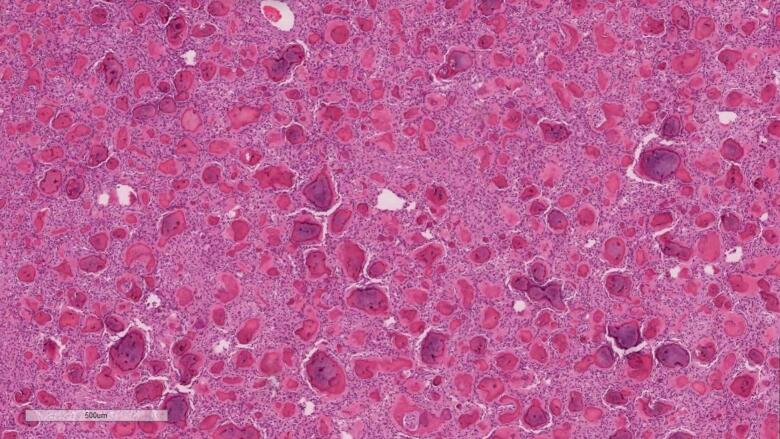
Hematoxylin & eosin stain magnification 5×. Low power view shows innumerable calcified bodies set within cellular stroma.

**Fig. 6:**
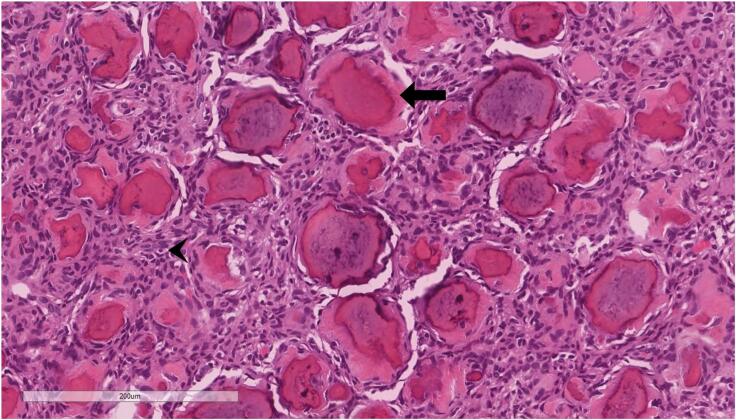
Hematoxylin & eosin stain magnification 20×. High power view demonstrates psammomatous calcifications surrounded by rim of collagenized material (arrow). The background stroma is cellular (arrowhead). Osteoblastic rimming is inconspicuous.

Microscopically in both masses, the lesion is composed of highly cellular fibrous stroma composed of spindled cells. Within the stroma, there is innumerable ossicles (calcifications) resembling psammoma bodies without osteoblastic rimming (Fig. 5, Fig. 6).

After nasal endoscopy and excisional biopsy of the nasal mass, the patient was doing well, with mild dizziness but no bleeding. She was tolerating oral intake, and no blurred vision was noted.

On examination, she was conscious, alert, and oriented. Her vital signs were stable, and she was afebrile. No diplopia or epistaxis was observed, and there was no bleeding from the posterior pharyngeal wall. The plan was to discharge the patient, with postoperative care and medications instructed. Lastly, emergency room instructions were provided.

## 3 Discussion

Ossifying fibromas are described as benign bony tumors that consist of highly cellular tissues with varying degrees of calcified tissue resembling bone [10]. The term “ossifying fibroma” has been in use since 1927 [11]. Later, a more detailed classification was established by the World Health Organization (WHO) in 1971, which categorized cementum-containing lesions into ossifying fibroma, fibrous dysplasia, cementifying fibroma, and cemento-ossifying fibroma. The WHO further classified benign fibro-osseous lesions into osteogenic neoplasms and non-neoplastic lesions of the bone [12].

The appearance of ossifying fibroma lesions in the nasal cavity is uncommon. In the literature, most cases of ossifying fibroma are typically found in sites such as the mandible and maxilla [4,5], followed by other locations like cranial and facial bones. Some cases have reported microscopically identical neoplasms in areas near the nasal cavity, such as the paranasal sinuses and nasopharynx. These cases exhibited cementum-like differentiation that is similar, but not identical, to ossifying fibroma [[13], [14], [15], [16]].

As most ossifying fibromas are found in the mandible or maxilla, they usually present as a painless bony mass that can give rise to symptoms depending on the site affected, such as displacement of adjacent teeth or root resorption [6]. The main reason for the appearance of symptoms is the direct expansion of the tumor itself, causing bone swelling and expansion that can affect the buccal and/or lingual endplates [6,[17], [18], [19]]. These lesions are often asymptomatic until they grow large enough to produce noticeable swelling and deformity. Smaller lesions are frequently discovered accidentally. In our case, the patient primarily had an ossifying fibroma located in the nasal cavity and maxilla, which led to symptoms such as nasal obstruction. Additionally, ossifying fibromas usually manifest in females during the second to fourth decades of life [17,18]. In our case, the patient was a 25-year-old female, indicating that the lesion is likely benign, as aggressive juvenile ossifying fibroma typically affects individuals younger than 15 years of age [20].

The importance of reporting cases such as ossifying fibroma of the nasal cavity is not to only think about common differential diagnoses of sinonasal masses/tumors, but to also think about other causes of sinonasal masses\tumors that are not commonly encountered and maybe missed or confused with other more common pathologies. Since ossifying fibroma is a bony lesion, CT scan of the paranasal sinuses was obtained. Additionally in our case, MRI scan of the paranasal sinuses was obtained to rule out soft tissue involvement (brain/orbital extension).

The management of ossifying fibroma is primarily surgical due to the tendency of these lesions to recur or possibly transform into malignant lesions if not excised. Literature suggests that without complete removal of the lesion, recurrence can occur between 6 months to 7 years after resection [21]. In our case, after examining the nasal mass, multiple biopsies were taken, and a complete excision of the mass was performed, leaving only a thin layer of the medial part of the middle turbinate. Furthermore, using a debrider, the nasal cavity and the mass were thoroughly debrided, leaving the nasal cavity and the maxillary sinus patent. Moreover, in many situations surgeons will remove diseased sinonasal mucosa without sending samples for histopathological investigations, as it is crucial to send any suspicious mass for histopathology since the management options differ depending on the pathology.

## 4 Conclusion

Ossifying fibroma is a benign bony lesion that most commonly affects the mandible and maxilla. It primarily occurs in females during their second to fourth decades of life. Such lesions are usually asymptomatic until they grow large enough to cause symptoms. In this case, we encountered an ossifying fibroma in the nasal cavity, which was managed surgically with complete resection. It is important to be aware of the radiologic findings of such lesions to facilitate early intervention and complete excision, thereby reducing the possibility of recurrence in the future.

## Consent

Written informed consent was obtained from the patient for publication and any accompanying images. A copy of the written consent is available for review by the Editor-in-Chief of this journal on request.

## Ethical approval

IRB review and approval was waived for this case report.

## Sources of funding

No funding was provided for the completion of this manuscript.

## Declaration of competing interest

There are no conflicts of interest to disclose.
